# Transition-Metal-Free C(sp^3^)–H Oxidation of Diarylmethanes

**DOI:** 10.3390/molecules23081922

**Published:** 2018-08-01

**Authors:** Fan Yang, Bihui Zhou, Pu Chen, Dong Zou, Qiannan Luo, Wenzhe Ren, Linlin Li, Limei Fan, Jie Li

**Affiliations:** 1 Department of Pharmacy, School of Medicine, Zhejiang University City College, No. 48, Huzhou Road, Hangzhou 310015, China; yfvskd@zju.edu.cn (F.Y.); 18868816170@163.com (B.Z.); cp15988801862@163.com (P.C.); lijie198455@126.com (D.Z.); luooooooo@163.com (Q.L.); a71109005@163.com (W.R.); lill@zucc.edu.cn (L.L.); fanlm@zucc.edu.cn (L.F.); 2 College of Pharmaceutical Sciences, Zhejiang University, Hangzhou 310058, China

**Keywords:** metal-free, diarylmethane, oxidation, ketone

## Abstract

An efficient direct C(sp3)–H oxidation of diarylmethanes has been demonstrated by this study. This method employs environment-friendly O_2_ as an oxidant and is promoted by commercially available MN(SiMe_3_)_2_ [M = K, Na or Li], which provides a facile method for the synthesis of various diaryl ketones in excellent yields. This protocol is metal-free, mild and compatible with a number of functional groups on substrates.

## 1. Introduction

The oxidation reaction is one of the most important transformations in organic synthesis by which oxygenated products of hydrocarbons were prepared and these compounds are valuable structural core in chemical and pharmaceutical industries [1]. Of these transformations, direct oxidation of methylene group of arylalkanes to ketones have attracted comprehensive attention, as diverse aryl ketone motifs are important structural units in numerous pharmaceuticals, naturally occurring molecules and organic functional materials [2,3,4,5,6]. Besides the traditional oxidation using KMnO_4_ as oxidant [7,8,9,10], advances in the synthetic method of aryl ketones were mainly based on three approaches: (1) classical Friedel-Crafts acylation of arenes [11], (2) oxidation of secondary alcohols [12] and CO insertion reactions [13,14]. Recently, significant progress has been made for the formation of aromatic ketones by transition-metal catalyzed oxidation of alkylarenes [15,16,17]. Notably, the latter represent a powerful tool in organic synthesis, while inevitably they will suffer from the use of toxic or expensive metals and ligands, harsh reaction conditions and generation of metal waste in most cases. Obviously, it is desirable to develop more practical protocols to achieve the synthesis of aryl ketones without the use of corrosive metal catalysts, hazardous stoichiometric oxidants and reductants [18,19,20,21]. Therefore, transition-metal-free methods for oxidation of alkylarenes are desirable in the pharmaceutical industry which can avoid the use of the heavy metal. For oxidation process, Molecular oxygen (O_2_) represents one of the best choices because of its low cost and has attracted substantial attention [22,23,24,25,26,27,28,29,30,31,32,33]. Herein, we wish to report the direct oxidation of diarylmethanes to diaryl ketones using O_2_-mediation by MN(SiMe_3_)_2_ [M = K, Na or Li], which represent a green and efficient synthetic method for this transformation.

## 2. Results

Initially, we commenced the reaction studies using 4-benzylpyridine (**1a**) as the model substrate. The control experiment was performed by stirring 4-benzylpyridine (**1a**) in THF under O_2_ at 60 °C for 16 h and no reaction occurred (entry 1, Table 1). To optimize the reaction conditions, various parameters such as temperature, bases, solvents were investigated. The strong bases play an important role in this transformation. As indicated in Table 1, the silylamides [MN(SiMe_3_)_2_, M = Li, Na, K] overwhelmed other bases (entries 2–8), giving the oxidative product in 76–85% yields. We anticipated that the inert sp^3^ C–H bond was transferred to carbanion before the direct insertion of O_2_, which needs the strong bases to achieve the deprotonation step. The solvent was next examined using LiN(SiMe_3_)_2_ as a base. Of the six solvents screened, THF showed the best performance (entries 4 and 9–13). Further screen of the reaction temperature indicated 60 °C is most appropriate. Only 35% yield of oxidation product **2a** was obtained at 40 °C after 12 h (entry 14), while no elevated yield was observed when the reaction was conducted in higher temperature 80 °C (entry 15). Furthermore, there is no oxidation product if the O_2_ was replaced with N_2_.

**Figure 1:**
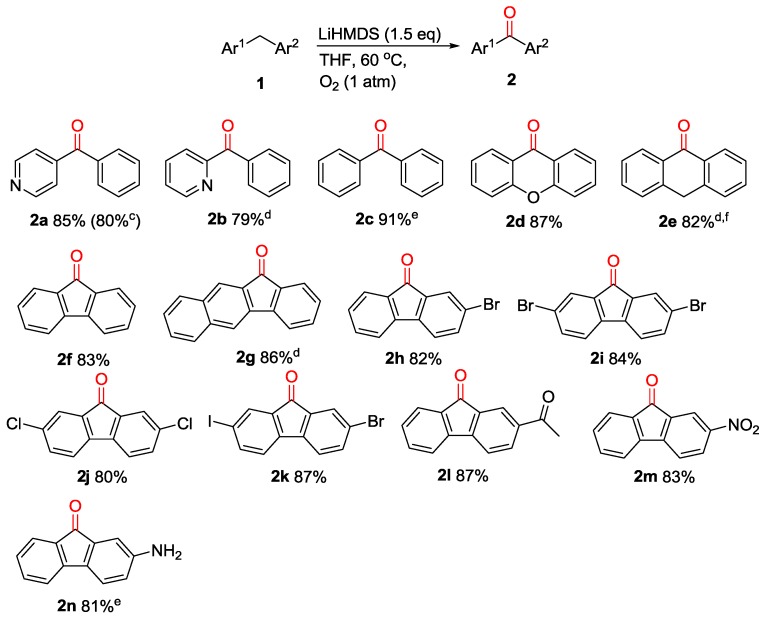
Scope of diarylmethanes in metal-free oxidation ^a,b^. ^a^ Reactions were conducted on a 0.1 mmol scale using 1 equiv of 1, 1.5 equiv of LiN(SiMe_3_)_2_ at 0.1 M. ^b^ Isolated yield. ^c^ Reaction conducted on 5 mmol scale. ^d^ LiHMDS was replaced with NaHMDS. ^e^ LiHMDS was replaved with KHMDS. ^f^ 80 °C.

With the optimal condition in hand, we then turn our attention to investigate the generality of this protocol. As presented in Figure 1, a variety of diarylmethanes were subjected to these reaction conditions. These reactions were conducted at 60 °C, except where noted. Diarylmethanes with different electronic and steric properties, such as 2-benzylpyridine (**1b**), diphenylmethane (**1c**), xanthene (**1d**), 9,10-dihydroanthracene (**1e**) and fluorene (**1f**), were proved to be good substrates, with corresponding products isolated in 79–91% yields. The family of fluorene analogues bearing various functional groups was examined next. The carbon-halo (electron-withdrawing) groups were compatible with this procedure and the oxidation products were obtained in excellent isolated yields (**2h**, **2i**, **2j**, **2k**). Additionally, remarkable chemoselectivity is observed with fluorene derivatives containing acetal, nitro and amino moieties, which all underwent oxygenation delivering the corresponding functionalized products (**2l**, **2m**, **2n**) in 81–87% yields.

To illustrate the scalablitlity of this methodology, we commenced the oxidation reaction of 4-benzylpyridine (**1a**) on a 5 mmol scale. The oxidation product **2a** was isolated in 80% yield (0.73 g).

## 3. Materials and Methods

### 3.1. General

^1^H- and ^13^C-NMR spectra were obtained on Bruker AVANCE III 500 MHz and 600 MHz spectrometers (Bruker Co., Billerica, MA, USA) with TMS as the internal standard; MS spectra were measured on a Finnigan LCQDECA XP instrument and an Agilent Q-TOF 1290 LC/6224 MS system (Santa Clara, CA, USA); silica gel GF_254_ and H (10–40 mm, Qingdao Marine Chemical Factory, Qingdao, China) were used for TLC and CC. Unless otherwise noted, all reactions were carried out under an atmosphere of oxygen.

### 3.2. Representative Procedure for the Oxidation of Diarylmethane

To an 8 mL oven-dried vial, 4-benzylpyridine (0.1 mmol), dry THF (1 mL), LiHMDS (0.15 mmol) were added subsequently. The reaction system was sealed by a rubber septum with a needle connected with O_2_ balloon. After stirring at 60 °C for 12 h, the reaction mixture was passed through a short pad of silica gel and eluted with ethyl acetate (1 mL × 3). The combined organics were concentrated under reduced pressure. The residue was purified by flash chromatography to give the diarylketone **2a** as white solid (15.6 mg, 85% yield). ^1^H NMR (500 MHz, CDCl_3_) *δ* 7.65 (d, *J* = 7.3 Hz, 2H), 7.54–7.43 (m, 4H), 7.33–7.25 (m, 2H). The ^1^H NMR data of **2a** was identical to those reported in the literature [34].

Analogous compounds **2b**–**n** were prepared according to the similar procedure for **2a**. **2b**: ^1^H NMR (500 MHz, CDCl_3_) *δ* 8.73 (d, *J* = 4.1 Hz, 1H), 8.10–8.03 (m, 3H), 7.90 (td, *J* = 7.8, 1.7 Hz, 1H), 7.60 (t, *J* = 7.4 Hz, 1H), 7.52–7.44 (m, 3H). The ^1^H NMR data of **2b** was identical to those reported in the literature [34]. **2c**: ^1^H NMR (500 MHz, CDCl_3_) *δ* 7.72 (dd, *J* = 5.2, 3.2 Hz, 2H), 7.54–7.47 (m, 1H), 7.43–7.36 (m, 2H). The ^1^H NMR data of **2c** was identical to those reported in the literature [35]. **2d**: ^1^H NMR (500 MHz, CDCl_3_) δ 8.34 (d, *J* = 7.9 Hz, 2H), 7.73 (t, *J* = 7.7 Hz, 2H), 7.49 (d, *J* = 8.4 Hz, 2H), 7.38 (t, *J* = 7.5 Hz, 2H). The ^1^H NMR data of **2d** was identical to those reported in the literature [36]. **2e**: ^1^H NMR (500 MHz, CDCl_3_): *δ* 8.26–8.37 (m, 2H), 7.56 (t, *J* = 7.4 Hz, 2H), 7.36–7.47 (m, 4H), 4.32 (s, 2H). The ^1^H NMR data of **2e** was identical to those reported in the literature [37]. **2f**: ^1^H NMR (500 MHz, CDCl_3_) *δ* 7.65 (d, *J* = 7.3 Hz, 2H), 7.54–7.43 (m, 4H), 7.33–7.25 (m, 2H). The ^1^H NMR data of **2f** was identical to those reported in the literature [36]. **2g**: ^1^H NMR (500 MHz, CDCl_3_): *δ* 8.14 (s, 1H), 7.88 (s, 1H), 7.85 (d, *J* = 7.5 Hz, 1H), 7.80 (d, *J* = 10.50 Hz, 1H), 7.73 (d, *J* = 9.5 Hz, 1H), 7.69 (d, *J* = 9.5 Hz, 1H), 7.75–7.55 (m, 2H), 7.45 (t, *J* = 9.2 Hz, 1H), 7.32 (t, *J* = 9.2 Hz, 1H). The ^1^H NMR data of **2g** was identical to those reported in the literature [38]. **2h**: ^1^H NMR (500 MHz, CDCl_3_): *δ* 7.78 (s, 1H), 7.68 (d, *J* = 7.3 Hz, 1H), 7.62 (d, *J* = 7.8 Hz, 1H), 7.53–7.52 (m, 2H), 7.41 (d, *J* = 7.8 Hz, 1H), 7.35–7.34 (m, 1H). The ^1^H NMR data of **2h** was identical to those reported in the literature [39]. **2i**: ^1^H NMR (500 MHz, CDCl_3_): *δ* 7.66 (d, *J* = 1.8 Hz, 2H), 7.52 (dd, *J* = 7.9 Hz, 1.8 Hz, 2H), 7.28 (d, *J* = 7.9 Hz, 2H). The ^1^H NMR data of **2i** was identical to those reported in the literature [39]. **2j**: ^1^H NMR (500 MHz, CDCl_3_) δ 7.62 (d, *J* = 1.8 Hz, 2H), 7.47 (dd, *J* = 8.0, 2.0 Hz, 2H), 7.44 (d, *J* = 7.9 Hz, 2H). The ^1^H NMR data of **2j** was identical to those reported in the literature [40]. **2k**: ^1^H NMR (500 MHz, CDCl_3_) *δ* 7.96 (d, *J* = 1.6 Hz, 1H), 7.84 (dd, *J* = 7.8, 1.6 Hz, 1H), 7.76 (d, *J* = 1.8 Hz, 1H), 7.63 (dd, *J* = 7.9, 1.9 Hz, 1H), 7.39 (d, *J* = 7.9 Hz, 1H), 7.28 (s, 1H). The ^1^H NMR data of **2k** was identical to those reported in the literature [41]. **2l**: ^1^H NMR (500 MHz, CDCl_3_) *δ* 8.17 (d, *J* = 0.98 Hz, 1H), 8.13 (dd, *J* = 7.8, 2.0 Hz, 1H), 7.70 (d, *J* = 7.3 Hz, 1H), 7.63–7.51 (m, 3H), 7.40–7.35 (m, 1H), 2.63 (s, 3H). The ^1^H NMR data of **2l** was identical to those reported in the literature [42]. **2m**: ^1^H NMR (500 MHz, CDCl_3_): *δ* 8.48 (d, *J* = 1.9 Hz, 1H), 8.43 (dd, *J* = 1.9 Hz, 8.2 Hz, 1H), 7.77 (d, *J* = 7.3 Hz, 1H), 7.72–7.67 (m, 2H), 7.62 (t, 1H), 7.46 (t, 1H). The ^1^H NMR data of **2m** was identical to those reported in the literature [39]. **2n**: ^1^H NMR (500 MHz, CDCl_3_) *δ* 7.49 (d, *J* = 7.3 Hz, 1H), 7.43–7.23 (m, 2H), 7.20 (d, *J* = 7.0 Hz, 1H), 7.16–7.00 (td, *J* = 7.2 Hz, 1.2 Hz, 1H), 6.89 (d, *J* = 2.3 Hz, 1H), 6.65 (dd, *J* = 7.9, 2.3 Hz, 1H), 3.82 (s, 2H). The ^1^H NMR data of **2n** was identical to those reported in the literature [43].

## 4. Conclusions

In conclusion, we have developed a metal-free, environmentally benign method for C(sp^3^)–H oxidation of various diarylmethanes using silylamides [MN(SiMe_3_)_2_, M = Li, Na, K] as base and O_2_ as an oxidant. This protocol provides a complementary method to prepare diaryl ketones in good to excellent yields. The detailed mechanism study is still underway.

## Figures and Tables

**Table 1:**
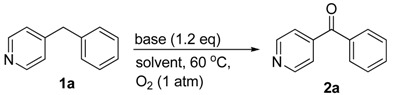
Optimization of the reaction conditions.

Entry ^a^	Base	Solvent	Temp [°C]	Yield [%] ^b^
1	–	THF	60 °C	–
2	KHMDS	THF	60 °C	76
3	NaHMDS	THF	60 °C	79
4	LiHMDS	THF	60 °C	85
5	KO*^t^*Bu	THF	60 °C	11
6	NaO*^t^*Bu	THF	60 °C	6
7	LiO*^t^*Bu	THF	60 °C	trace
8	CS_2_CO_3_	THF	60 °C	–
9	LiHMDS	dioxane	60 °C	23
10	LiHMDS	toluene	60 °C	15
11	LiHMDS	DME	60 °C	79
12	LiHMDS	CPME	60 °C	56
13	LiHMDS	CH_2_Cl_2_	60 °C	11
14	LiHMDS	THF	80 °C	84
15	LiHMDS	THF	40 °C	35
16 ^c^	LiHMDS	THF	60 °C	–

^a^ Reactions were carried out using 4-benzylpyridine (0.1 mmol) and base (0.15 mmol) in anhydrous solvent (1.0 mL) under O_2_ for 12 h. ^b^ Isolated yields. ^c^ O_2_ was replaced with N_2_.
